# Hypoparathyroidism Causing Seizures: When Epilepsy Does Not Fit

**DOI:** 10.1155/2018/5948254

**Published:** 2018-04-01

**Authors:** Faheem Seedat, Reyna Daya, Sindeep A. Bhana

**Affiliations:** ^1^Department of Internal Medicine, Chris Hani Baragwanath Academic Hospital, Faculty of Health Sciences, University of the Witwatersrand, Johannesburg, South Africa; ^2^Division of Endocrinology and Metabolism, Department of Internal Medicine, Chris Hani Baragwanath Academic Hospital, Faculty of Health Sciences, University of the Witwatersrand, Johannesburg, South Africa

## Abstract

A 24-year-old man presented to the Chris Hani Baragwanath Academic Hospital emergency department with recurrent seizures having previously been diagnosed with epilepsy from age 14. The biochemical investigations and brain imaging were suggestive of seizures secondary to hypocalcemia, and a diagnosis of idiopathic hypoparathyroidism was confirmed. After calcium and vitamin D replacement, the patient recovered well and is seizure free, and off antiepileptic therapy. This case highlights the occurrence of brain calcinosis in idiopathic hypoparathyroidism; the occurrence of acute symptomatic seizures due to provoking factors other than epilepsy; and the importance, in the correct clinical setting, of considering alternative, and sometimes treatable, causes of seizures other than epilepsy.

## 1. Introduction

An adult onset seizure is a scenario often encountered by internists. Patients are often labeled as epileptic and in some cases, appropriate investigations to assess for provoking factors and/or treatable causes are overlooked. This case report highlights such a scenario when a patient with hypocalcemia-associated seizures due to underlying idiopathic hypoparathyroidism (IH) was treated for idiopathic epilepsy for a decade. We consider the entity of acute symptomatic seizures and a reasonable approach to investigating adults with new onset seizures. Furthermore, we highlight hypocalcemia as a cause for seizures and discuss the occurrence of brain calcinosis in IH and the role of brain calcinosis versus hypocalcemia as the aetiology of seizures in these patients.

## 2. Case Report

**Figure 1:**
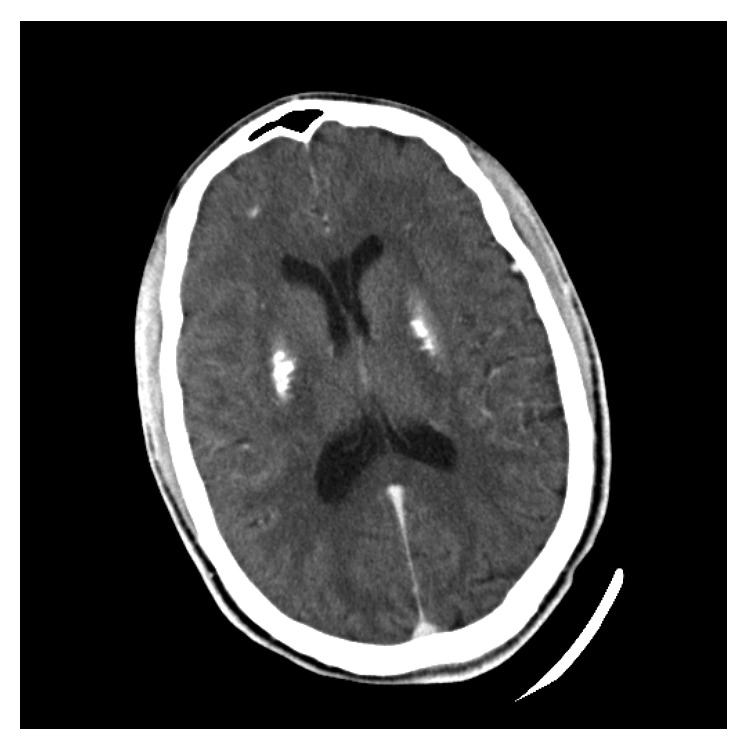
Calcification of the lentiform nucleus.

**Figure 2:**
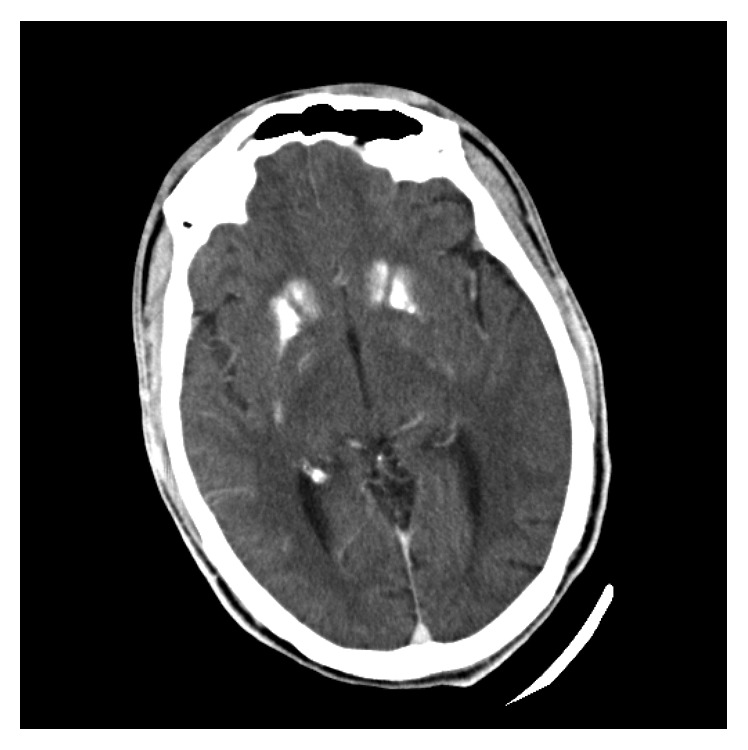
Calcification of the caudate nucleus.

**Figure 3:**
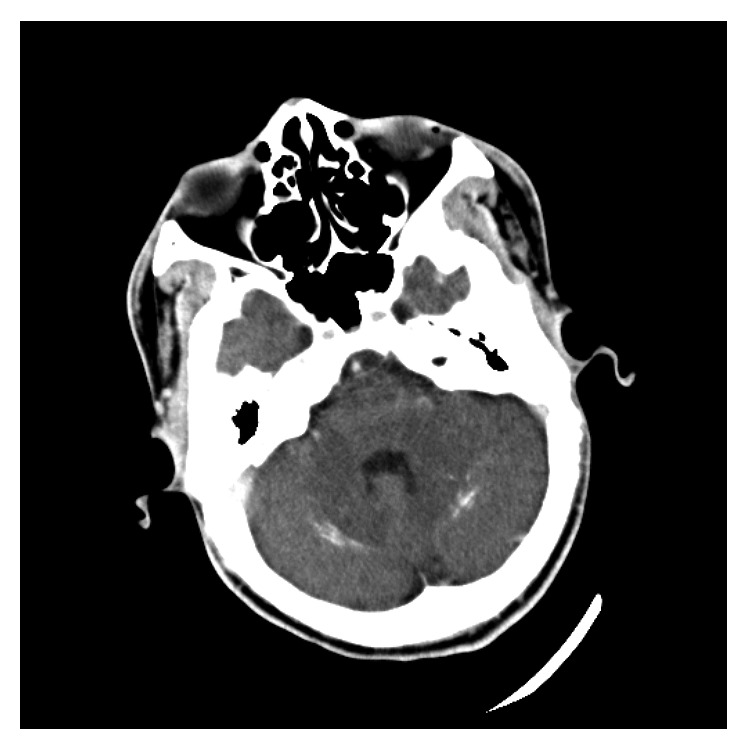
Cerebellar calcification.

A 24-year-old man presented to the emergency department with recurrent seizures preceded by a history of perioral and fingertip paresthesia. He was diagnosed with epilepsy at the age of 14 and treated with sodium valproate but denied a full diagnostic workup for seizures. On examination, both Chvostek's and Trousseau's signs were present. On biochemical investigations, his serum-corrected calcium measured 1.03 mmol/L (2.15–2.5 mmol/L), magnesium 0.72 mmol/L (0.63–1.05 mmol/l), and phosphate 2.57 mmol/L (0.78–1.42 mmol/L), and a total 25-OH vitamin D measured 55.98 nmol/L (deficient <50 nmol/L, insufficient 52.5–72.5 nmol/L, and sufficient >72.5 nmol/L). His serum parathyroid hormone was 0.6 pmol/L (1.6–6.9 pmol/L), despite vitamin D insufficiency, confirming a diagnosis of IH. A computed tomography (CT) brain scan showed calcification of the basal ganglia (Figures 1 and 2) and cerebellum (Figure 3). This patient was initially treated with an intravenous calcium infusion and subsequently started on chronic oral calcium supplementation along with alfacalcidol to maintain a serum calcium level in the low normal range. Sodium valproate was stopped as it was felt that his seizures were likely secondary to the profound hypocalcemia. At the 4-month follow-up visit, he was seizure free.

## 3. Discussion

The International League Against Epilepsy (ILAE) defines a seizure as a “*transient occurrence of signs and/or symptoms due to abnormal excessive or synchronous neuronal activity in the brain*” [1]. Epilepsy is characterized by a history of at least one seizure with a predisposition, due to an alteration in the brain, to generate future seizures and associated neurobiologic, cognitive, psychological, and social disturbances [1].

The terminology used to classify seizure aetiology has been extensively debated [2, 3]. The ILAE recommends a distinction between acute symptomatic seizures, referring to seizures occurring in “*close temporal relationship with an acute central nervous system (CNS) insult, which may be metabolic, toxic, structural, infectious or due to inflammation*,” and unprovoked seizures or epilepsy where no identifiable clinical condition responsible for the seizure is found or the time interval is beyond that estimated for acute symptomatic seizures [4].

In the adult presenting with definite new onset seizures, guidelines suggest initial investigations for any provocative factors that may cause acute symptomatic seizures. Workup should begin with a thorough bedside clinical evaluation and further investigations tailored to the initial clinical findings, for example, neuroimaging in the presence of a suspected structural brain lesion. Due to cost-efficacy and accessibility, CT scan is recommended as the initial imaging modality; however, some abnormalities may be missed by CT, and if concerns still exist for a structural lesion, magnetic resonance imaging is suggested [5, 6]. Evidence for the investigation of biochemical abnormalities or drug intoxication responsible for seizures is conflicting. Studies supporting routine laboratory or radiological testing are biased by being performed in the emergency department where the prevalence of acute symptomatic seizures is high compared to studies done outside the emergency department where the prevalence of biochemical abnormalities ranges from 0 to 15%, but often these abnormalities are not of clinical relevance. As such, it is recommended that the use of laboratory testing be guided by clinical features suggesting a provoking factor, such as in our patient, who complained of a history of perioral and fingertip parethesias, and hence, a serum calcium measurement was appropriate [5, 6]. No studies have examined the use of a lumbar puncture in the work-up of new onset seizures and, once more, this should be guided by the clinical evaluation; in the South African setting, this is a necessary investigation in HIV-positive patients presenting with seizures due to the high risk of CNS infections in this population [5, 6]. Electroencephalography (EEG) should be used in patients where neurological function has not improved within 30–60 minutes after the end of the seizure and in those with a fluctuating level of consciousness or when focal neurological signs are not explained by a structural lesion [5, 6].

The aetiology of acute symptomatic seizures is divided into two groups: neurological insults and systemic diseases. Neurological insults comprise just under half of all cases and include acute stroke (16%), traumatic brain injuries (16%), and CNS infections (15%). Systemic diseases include electrolyte and metabolic disorders (9%); medications, drugs, and toxins (14%); anoxic encephalopathy and limbic encephalitis (5%); and eclampsia (2%) [4, 7]. In sub-Saharan Africa, particular common causes of neurological-related acute symptomatic seizures include the following: CNS infections such as neurocysticercosis, herpes simplex virus encephalitis, HIV-related opportunistic infections, traumatic brain injury, and cerebrovascular accidents [8, 9].

The electrolyte abnormalities associated with seizures include hyper- and hyponatremia, hyper- and hypocalcemia, and hypomagnesemia. According to the ILAE, to attribute a seizure to an electrolyte abnormality, the biochemical derangement should be detected within 24 hours of the seizure [4, 10]. Hypocalcemic-related seizures depend on the rate of decrease in serum calcium and degree of hypocalcemia [10]. The resultant hypocalcemia results in increased neuronal excitability due to reduced extracellular concentration of calcium rather than depleted intracellular levels and both generalised tonic-clonic or focal motor seizures can occur with hypocalcemia [10, 11].

IH is a well-known cause of hypocalcemia. Acute symptomatic seizures due to electrolyte abnormalities do not have structural brain lesions; however, IH is an exception as brain calcification or calcinosis, which was first described by Eton in 1939, can occur [10, 12, 13]. Despite high rates of brain calcinosis in most patients with IH, between 73% and 93%, the pathogenesis remains unknown. It is postulated that brain calcinosis occurs due to a milieu of poor calcium control and a high calcium-phosphate product, particularly in the cerebrospinal fluid, resulting in calcium deposition predominantly in the periventricular regions [13]. The basal ganglia, in particular the lentiform (putamen and globus pallidus) and caudate nuclei, are the most frequently affected sites although more extensive intracerebral calcification affecting other brain parenchymal structures, as in this patient, may occur [12, 13]. The degree of brain calcification is directly related to the severity and duration of hypocalcemia, with a critical duration of illness exceeding 4 years before the occurrence of calcification and the odds of brain calcinosis increasing by 12% for every year of hypocalcemic symptoms [13].

The clinical presentation of brain calcification is varied and includes the following: seizures, mental deterioration, and movement disorders from cerebellar and extrapyramidal dysfunction [12, 13]. In IH, seizures are a significant predictor of the existence and progression of brain calcinosis, but the odds of epilepsy are only 0.9% suggesting the aetiology for the seizures appears not to be related to the underlying brain calcinosis but rather due to neuronal excitability from low levels of calcium [13]. In an Indian cohort, the prevalence of seizures in IH was 64%, and just below 90% presented with a generalised tonic-clonic seizure, although partial complex seizures did also occur (4.4%) [14]. Basal ganglia calcification is easily seen on CT scan, as in our patient; however, nuclear imaging modalities such as ^18^F-FDG may also be used to confirm basal ganglia calcification through reduced basal ganglia glucose uptake, which is directly proportional to the degree of basal ganglia calcification [15].

Calcium and vitamin D supplementation is the recommended therapy for IH, although this has not been shown to slow the progression of brain calcinosis. The serum calcium is maintained in the lower range of normal to avoid hypercalciuria and resultant nephrolithiasis [13]. Regarding the management of seizures in IH, antiepileptic drugs (AEDs) are used; in an Indian cohort of patients with IH and seizure, most patients only required a single agent (71%) of which phenytoin was most commonly used (46.7%), followed by valproate (40%), carbamazepine (26.7%), and levetiracetam (13.3%) [14]. In this study, AEDs were continued for 2 years after the patient was seizure free and only discontinued when the patient had a normal EEG and an average serum calcium level greater than 1.8 mmol/L for the last three clinic visits [14]. When AEDs were withdrawn, the first drug withdrawn was the first drug started and the dose was reduced by 25% each month [14]. The effect of AEDs on calcium absorption and vitamin D metabolism has concerning effects on calcium homeostasis. In an Indian study, the discontinuation of phenytoin, valproate, and carbamazepine was all associated with increased serum calcium levels, and the effects of AEDs on calcium levels must be taken into account when considering calcium and vitamin D replacement in IH [14].

## 4. Conclusion

This case highlights acute symptomatic seizures, which are caused by disease processes other than epilepsy, and highlights the need, in the correct clinical setting, to consider other treatable differential diagnosis for seizures. In this patient, despite a prior diagnosis of epilepsy, he now remains seizure free and off antiepileptic drugs. Furthermore, we highlight the entity of brain calcinosis in hypoparathyroidism, a diagnosis that should be considered when these radiological features are seen on a brain CT scan.
